# Characterization and In Vitro Evaluation of Porous Polymer-Blended Scaffolds Functionalized with Tricalcium Phosphate

**DOI:** 10.3390/jfb15030057

**Published:** 2024-02-26

**Authors:** Iwona Pudełko-Prażuch, Mareeswari Balasubramanian, Sundara Moorthi Ganesan, Stanisław Marecik, Kamila Walczak, Kinga Pielichowska, Suvro Chatterjee, Ravichandran Kandaswamy, Elżbieta Pamuła

**Affiliations:** 1 Department of Biomaterials and Composites, Faculty of Materials Science and Ceramics, AGH University of Krakow, Al. Mickiewicza 30, 30-059 Krakow, Poland; ipudelko@agh.edu.pl (I.P.-P.); smarecik@agh.edu.pl (S.M.); kamwalczak@agh.edu.pl (K.W.); kingapie@agh.edu.pl (K.P.); 2 Department of Rubber and Plastics Technology, Madras Institute of Technology Campus, Anna University, Chromepet, Chennai 600 044, Tamil Nadu, India; venibala18@gmail.com (M.B.); sundaramoorthi1997@gmail.com (S.M.G.); 3 Department of Biotechnology, Golapbag Campus, University of Burdwan, Burdwan 713 104, West Bengal, India; soovro@yahoo.ca

**Keywords:** PLA, polymer scaffolds, porous scaffolds, polymer blends, TCP, polymer functionalization

## Abstract

Bone tissue is one of the most transplanted tissues. The ageing population and bone diseases are the main causes of the growing need for novel treatments offered by bone tissue engineering. Three-dimensional (3D) scaffolds, as artificial structures that fulfil certain characteristics, can be used as a temporary matrix for bone regeneration. In this study, we aimed to fabricate 3D porous polymer scaffolds functionalized with tricalcium phosphate (TCP) particles for applications in bone tissue regeneration. Different combinations of poly(lactic acid) (PLA), poly(ethylene glycol) (PEG with molecular weight of 600 or 2000 Da) and poly(ε-caprolactone) (PCL) with TCP were blended by a gel-casting method combined with rapid heating. Porous composite scaffolds with pore sizes from 100 to 1500 µm were obtained. ATR-FTIR, DSC, and wettability tests were performed to study scaffold composition, thermal properties, and hydrophilicity, respectively. The samples were observed with the use of optical and scanning electron microscopes. The addition of PCL to PLA increased the hydrophobicity of the composite scaffolds and reduced their susceptibility to degradation, whereas the addition of PEG increased the hydrophilicity and degradation rates but concomitantly resulted in enhanced creation of rounded mineral deposits. The scaffolds were not cytotoxic according to an indirect test in L929 fibroblasts, and they supported adhesion and growth of MG-63 cells when cultured in direct contact.

## 1. Introduction

In recent years, bone tissue was the second most transplanted tissue after blood [1,2]. This growing demand for new solutions provided by bone tissue engineering is caused by common trauma or pathologies, different diseases, and the ageing population [3]. The aim of bone tissue engineering is to design biomaterials that temporarily mimic the three-dimensional structure and functions of bone to promote cell adhesion, proliferation, and differentiation [4].

Bone tissue has a very complex and highly organized structure. When it comes to its chemical composition, it consists of from 50% to 70% inorganic constituents (mainly hydroxyapatite), 20% to 30% organic constituents (type I collagen), 5% to 10% water, and 3% lipids. While if its architecture is taken into account, bone tissue can be classified as hard cortical bone (with a porosity of 10–30%) or spongy cancellous bone (with a porosity of 30–90%) [1,5]. Another important characteristic of cancellous bone tissue is its interconnected porous network with a high ratio of bone surface to bone volume [4].

Scaffolds are three-dimensional artificial structures that act as a temporary matrix providing an environment for bone regeneration and development [2]. First, scaffolds should be biocompatible, which means that they are supposed to perform their functions while maintaining a suitable host response. Nontoxicity is another important requirement, as they should not cause necrosis of cells. Second, scaffolds should have the ability to be actively degraded at a reasonable rate by hydrolysis or enzymatic activity throughout the regeneration process [6]. Degradation products should also not be toxic to cells. Lastly, porosity is a very important key factor, as it provides better fixation and osteointegration with the host tissues. The ideal scaffold for bone engineering would have interconnected porosity. The minimum requirement for the pore size is about 100 µm but ideally its size should fit in the range of 250–400 µm [7]. Although the size of osteoblasts ranges from 10 µm to 50 µm, they prefer larger pores (100–200 µm) as they allow macrophages to infiltrate and eliminate current pathogens. On the other hand, smaller pores (<100 µm) promote the formation of fibrous tissue and non-mineralized osteoid. Furthermore, pores smaller than 10 µm create a high surface-to-volume ratio and thus facilitate better ion exchange and bone protein adsorption [8]. At the same time, large pores promote blood supply and bone ingrowth [9]. In general, to achieve the best mimic of the bone tissue environment, both microporosity and macroporosity must be obtained.

Poly(lactic acid) (PLA) is considered to be one of the most versatile aliphatic polyesters. It has two chiral configurations, poly-L-lactic acid and poly-D-lactic acid, and their ratio allows one to optimize the degradation rate. PLA is considered a biodegradable material with a low rate of degradation, which means that it can remain in vivo for up to 5 years. Its breakdown occurs through a hydrolysis reaction and the rate of this process is determined by molecular weight and crystallinity [10]. In addition, PLA is considered to be a thermally stable, biocompatible, and non-inflammatory material with nontoxic degradation products [1]. However, due to features such as low heat resistance or low strength, its applications are restricted [11]. To overcome the limitations mentioned, it is possible to combine PLA with different materials, such as ceramics, or to blend it with other polymers [12,13]. Poly(ε-caprolactone) (PCL) belongs to the group of semicrystalline linear aliphatic polyesters. Because of its biocompatibility, biodegradability, and structural stability, it is commonly used in tissue engineering. However, low bioactivity and low surface energy lead to reduced cell affinity. Blending PLA with PCL allows adjustment of the degradation rate and mechanical properties and the obtainment of less hydrophobic materials [14,15]. Another example of a widely used polymer is poly(ethylene glycol) (PEG). It is a semicrystalline hydrophilic polyether, which is also considered to be a biocompatible, biodegradable, and nontoxic material. As it has fairly low glass-transition and melting temperatures, which increase with the molecular weight of PEG, it is commonly mixed with PLA [16]. The addition of PEG to PLA enhances flexibility and polarity, increases hydrophilicity, and allows the degradation of the materials to be better controlled [17]. Different methods are being used to create scaffolds from polymer blends, such as solvent casting, particle leaching, freeze drying, electrospinning, thermal-induced phase separation (TIPS) and 3D printing [18,19,20,21]. However, the method of gel-foam casting that we present is a simple, affordable, and effective technique that allows us to obtain scaffolds with different pore sizes and a porosity of approximately 60%.

The most biomimetic scaffolds are polymer-ceramic composites, as they can replicate the composition and architecture of the extracellular matrix of bone tissue [1,22]. Especially interesting nowadays are bioactive ceramics such as tricalcium phosphate (TCP). TCP has two crystalline forms: α-TCP and β-TCP [23,24]. The latter is more favored because of its closest similarity to bone tissue in terms of chemical composition and crystallinity. Fillers in the form of microparticles can be osteoconductive, which eventually leads to improved cell proliferation. Furthermore, TCP exhibits a rate of degradation similar to the rate of new bone formation [25,26]. The goal of this study was to obtain PLA–TCP composites with or without additives such as PEG600, PEG2000, and PCL and to assess their physicochemical and biological properties. In this research, we aimed to fabricate different polymer blends that exhibit great cytocompatibility with improved osteointegration and are characterized by different degradation rates. Blending PLA with different polymers can change the susceptibility to degradation, as well as changing wettability, which affects cell adhesion. Many researchers have studied PLA/TCP composites [23,27,28,29], as well as PCL/TCP composites [30,31,32,33,34]. Senatov et al. evaluated PLA/PCL scaffolds enriched with hydroxyapatite, showing their potential in regenerative medicine [35]. Furthermore, Liu et al. proposed PLA/TCP scaffolds containing PEG as an alternative for bone defect repair [36]. However, to the best of our knowledge the blends fabricated with the use of the method we provide have not been previously investigated or compared. We present five different compositions of polymer blends that are characterized by different degradation rates without significant changes in wettability. The scaffolds were fabricated with the use of a gel-foam casting method combined with rapid heating, and their properties were characterized. To do so, microscopy observations (scanning electron microscopy and optical microscopy), differential scanning calorimetry (DCS), and Fourier transformed infrared spectroscopy (FTIR) were performed, as well as measurement of water contact angle and cell culture tests. Based on the gathered information, it was possible to assess which composites are more suited to bone tissue-engineering applications while considering microstructure, cytocompatibility, and susceptibility to degradation.

## 2. Materials and Methods

### 2.1. Materials

Poly(lactic acid) (PLA, Ingeo Biopolymer 3052 D, M_n_ = 180,000) was obtained from Nature Works LLC, courtesy of Natur Tec Pvt Ltd., Chennai, Tamil Nadu, India. Poly(ε-caprolactone) (PCL, M_n_ = 80,000) and poly(ethylene glycol) (PEG, M_n_ = 600 and M_n_ = 2000) were purchased from Sigma-Aldrich, Steinheim, Germany. Beta-tricalcium phosphate (TCP, analytical reagent grade, particle size < 500 µm) was provided by Sisco Research Laboratories Pvt. Ltd (Sisco Labs, Mumbai, India). Dichloromethane and chloroform were purchased from Merck KGaA, Darmstadt, Germany. Phosphate buffered saline (PBS) was obtained from VWR Life Science, Radnor, PA, USA. The L929 mouse fibroblast cell line was provided by the American Type Culture Collections (Manassas, VA, USA) and the osteoblast-like MG-63 cell line derived from an osteosarcoma was provided by the European Collection of Cell Cultures (Salisbury, UK). Dulbecco’s modified eagle medium (DMEM), minimum essential medium (MEM), fetal bovine serum (FBS), penicillin and streptomycin mixture, amino acids, and sodium pyruvate for cell culture were purchased from PAN Biotech, Aidenbach, Germany. Calcein AM, propidium iodide, and resazurin were provided by Sigma-Aldrich, Steinheim, Germany.

### 2.2. Preparation of Scaffolds

Agel-foam-casting technique combined with rapid heating was used to fabricate scaffolds, as previously described [37]. First, predetermined quantities of all components (Table 1) were mixed and diluted in dichloromethane, while stirring on a magnetic stirrer at a speed of 300 rpm to achieve a homogeneous mixture. Subsequently, the solution was poured onto a glass plate preheated to 70 °C, which led to a rapid evaporation process, resulting in the creation of pores of different sizes. Since PLA is considered biocompatible and biodegradable and has been extensively studied for application in bone tissue [1,10,38], we used this semicrystalline polyester as a matrix for all evaluated blends. In order to enhance the osteoinduction and osteointegration of PLA, particles of β-TCP were incorporated. Moreover, the addition of ceramic filler can be used to modify the mechanical properties of manufactured scaffolds. Furthermore, the presence of PCL in the matrix may reduce the brittle behavior of PLA [39,40]. Interestingly, PEG has been reported to act as a plasticizer [41,42]. As β-TCP was the only inorganic filler used in this study, we preserved its percentage at the same level in each blend. To determine the impact of the molecular weight of PEG, the same amount of each (600 or 2000) was used in samples 2 (PLA-TCP-PEG2000) and 3 (PLA-TCP-PEG600).

### 2.3. Physicochemical Characterization of Composite Scaffolds

#### 2.3.1. Fourier Transform Infrared Spectroscopy

Attenuated total reflectance Fourier transform infrared spectroscopy (ATR-FTIR, Tensor 27, Bruker, Billerica, USA) in the 4000 to 500 cm^−1^ wavenumber range was used to investigate the composition of prepared scaffolds. To do so, cylinders with 12 mm diameter and 2 mm height were cut from the scaffolds and placed on the diamond crystal, and the spectra were recorded. The spectra were acquired by averaging 16 scans with a resolution of 1 cm^−1^. OPUS software (version 7.2, Bruker, Billerica, USA) was used to process the data.

#### 2.3.2. Differential Scanning Calorimetry

Differential scanning calorimetry (DSC, Mettler Toledo DSC1 calorimeter, Greifensee, Switzerland) was used to analyze the thermal properties of the prepared scaffolds. The samples (approx. 4 mg) were placed in pierced aluminum pans. The test was carried out in a nitrogen atmosphere (30 mL/min) in heating/cooling/heating cycles in a temperature range of −90 °C to 210 °C at a heating/cooling rate of 10 K/min.

#### 2.3.3. Optical and Scanning Electron Microscopy

The microstructure and morphology of the scaffolds were studied using an optical microscope (VHX-900F, Keyence, Mechelen, Belgium) and a scanning electron microscope (SEM, Aero S, Thermo Fisher Scientific, Waltham, MA, USA). Cylinders 12 mm in diameter and 1 mm in height were photographed with a digital camera and observed with the use of an optical microscope. For SEM, scaffolds were glued onto holders with carbon tape and sputter coated with a thin carbon layer to make them conductive, and observations of microstructure were made.

#### 2.3.4. Contact Angle Measurement

The water contact angle was measured to evaluate the wettability of the scaffolds. The syringe was filled with deionized water and the material was placed on a table in the camera’s field of view. Then, a 2 µL drop of MilliQ-water was placed on the surface of the materials at room temperature using a drop shape analyzer (DSA 10, KRÜSS GmbH, Hamburg, Germany) and the contact angle was evaluated by averaging 10 individual droplets.

#### 2.3.5. Degradation Studies

Degradation studies were conducted to evaluate the impacts of different additives on the degradation rate. To do so, the samples were immersed in phosphate buffered saline solution (PBS) in a ratio of 1:100 (1 g of sample per 100 mL of PBS) and incubated at 37 °C for up to 4 weeks. In the predetermined periods of time (1, 2, 3, and 4 weeks), samples were taken from the solution, rinsed with Milli-Q water, freeze dried and weighed to determine weight loss (WL). The WL was calculated using following Formula (1)

(1) $$WL=\frac{M_{0}-M_{i}}{M_{0}}\cdot 100\%$$

where

$M_{0}$—initial mass of the sample [g];

$M_{i}$—mass of the sample after degradation [g].

### 2.4. Biological Evaluation

#### 2.4.1. Studies on Extracts

To verify the cytotoxicity of the scaffolds obtained, a study of L929 fibroblasts incubated with the extracts was carried out. Briefly, we immersed the samples in culture medium (1 g of sample per 10 mL of medium) and the extraction process was carried out for 24 h at 37 °C. Cells, in amounts of 10,000 per well, were seeded in a 96-well plate (Sarstedt, Nümbrecht, Germany) and cultured for 24 h at 37°C in a 5.0% CO_2_ atmosphere in DMEM supplemented with fetal bovine serum (FBS, 10%) and antibiotics (mixture of penicillin and streptomycin, 1%). After 24 h, the medium was replaced with extracts obtained previously and culturing was carried out for 1 and 4 days.

The AlamarBlue metabolic assay and live/dead staining were performed on days 1 and 4 after the addition of the extracts. To investigate metabolic activity, 150 µL of 10% AlamarBlue solution in DMEM was added to each well and the plate was incubated for 4 h at 37 °C in a 5.0% CO_2_ atmosphere. Then, 100 µL of the solution was transferred to a black 96-well plate (Thermo Fisher Scientific, Waltham, MA, USA) to measure fluorescence (λ_ex_ = 544 nm and λ_em_ = 590 nm, FluoStar Omega, BMG Labtech, Ortenberg, Germany). The range used to calculate the reduction of resazurin was 0% for the reagent incubated in an empty well and 100% for the reagent reduced in autoclave, and the values were compared to the control sample, which was cells cultured in supplemented DMEM as previously described [43,44].

Calcein AM and propidium iodide (0.1% *v*/*v* each) were mixed with PBS to prepare the live/dead reagent. After removing the medium from the wells, 100 µL of the reagent was added to each well and incubated for 20 min in darkness. Subsequently, with the use of a fluorescent microscope (ZEISS Axiovert 40 CFL with metal halide illuminator, Oberkochen, Germany), pictures of live and dead cells were taken.

#### 2.4.2. In Vitro Cytocompatibility

Osteoblast-like MG-63 cells were used to assess the cellular response of scaffolds. To do so, scaffolds of 12 mm diameter and 2 mm height were placed in a 24-well plate. Samples were sterilized by immersing them in 70% ethanol for 2 h, followed by UV irradiation on both sides for 20 min each. Then, on each scaffold we seeded 20,000 cells suspended in 1 mL MEM supplemented with fetal bovine serum (FBS, 10%), antibiotics (mixture of penicillin and streptomycin, 1%), amino acids (0.1%) and sodium pyruvate (0.1%). Cells were cultured for 1, 3, or 7 days at 37 °C in a 5.0% CO_2_ atmosphere. At each time point, the AlamarBlue assay and live/dead staining were performed as described in Section 2.4.1. For AlamarBlue, 1 mL resazurin reagent was added to each well, and the cells were incubated for 4 h. To measure fluorescence, 100 µL was transferred to each well of the black 96-well plates for fluorescence measurement. For live/dead staining, we used 0.5 mL of the respective reagent per well. After incubation in darkness, the samples were taken from the plate, placed on the microscope slide, and pictures were taken as described above.

### 2.5. Statistical Analysis

All data are expressed as the average values ± standard deviation (SD) of two independent experiments performed in triplicate. Normal distribution was verified using the Shapiro–Wilk test followed by a one-way analysis of variance (ANOVA) test with the LSD Fisher post hoc test to determine statistical significance. Origin software (version 2022 SR1, OriginLab Corporation, Northampton, MA, USA) was used and we considered a probability value of less than 0.05 as statistically significant: * represents *p* < 0.05, ** represents *p* < 0.01, and *** represents *p* < 0.001.

## 3. Results

### 3.1. ATR-FTIR Spectroscopy

**Figure 1:**
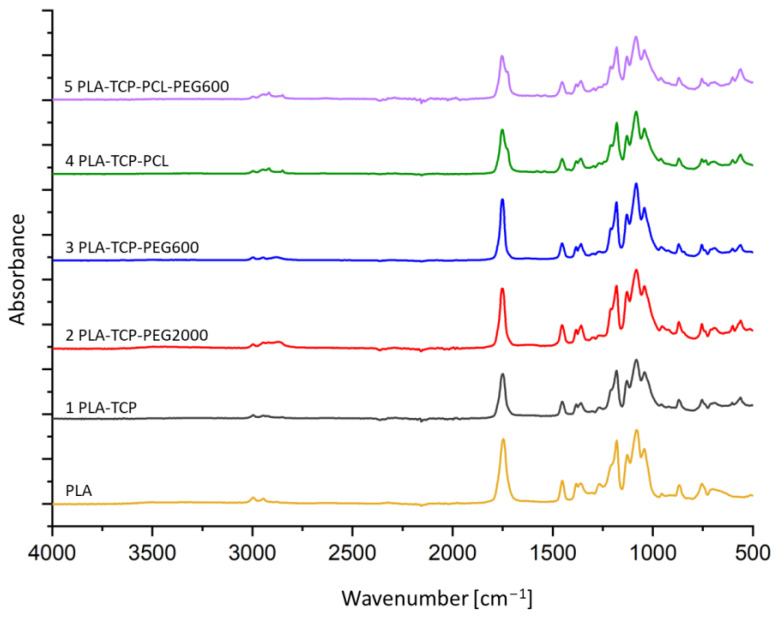
FTIR spectra of composite scaffolds.

ATR-FTIR was performed to identify the chemical bonds of the components of the fabricated scaffolds. Figure 1 shows the spectra of all samples. Each spectrum has a band around 1750 cm^−1^, resulting from the C=O stretching vibrations of PLA [45,46]. However, the double peak in this range suggests the presence of a component other than PLA. It is visible in samples containing PCL, which confirms that both polymers (PLA and PCL) are blended [45,47]. Bands in the range 2800–3000 cm^−1^ originate from C-H stretching vibrations of CH_3_ groups in PLA and from CH_2_ groups from PCL and PEG (both 600 and 2000) [45]. The presence of bending vibrations of the C-H groups is reflected by the bands in the range 1500–1250 cm^−1^. Bands around 1450 and 1370 cm^−1^ are assigned to symmetric and antisymmetric bending vibrations of the -CH_3_ groups, respectively, which originate from PLA [46]. The bands located between 1200 and 1000 cm^−1^ are due to C-O stretching vibrations derived from all polymer components of the investigated samples [45,46,47]. Bands at around 600 cm^−1^ that are not present in the PLA spectrum correspond to the vibration of PO_4_^3−^ groups derived from β-TCP [48,49]. All of the spectra have bands at similar wavenumbers and intensities, which suggests that no new chemical bonds were formed during the preparation of the blends.

### 3.2. Differential Scanning Calorimetry

**Figure 2:**
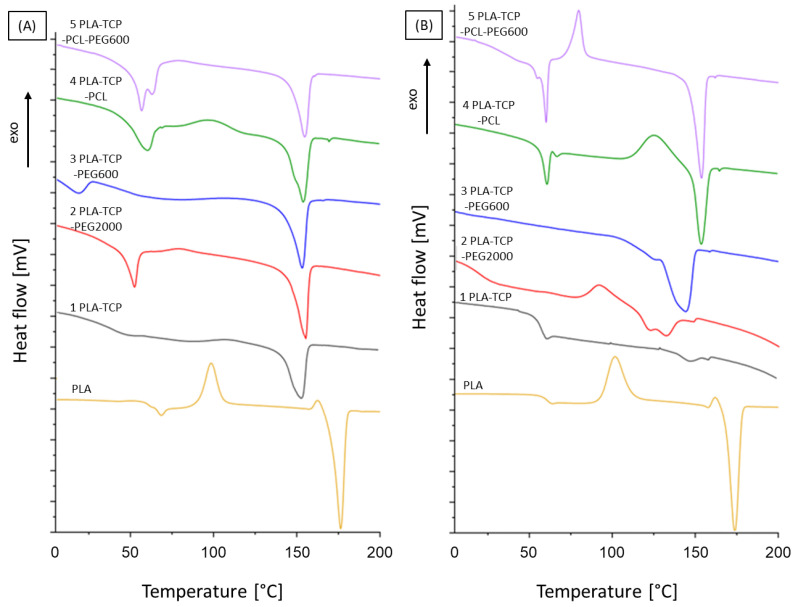
DSC results for the evaluated samples: (**A**) first heating cycle; and (**B**) second heating cycle.

DSC analysis was performed to determine the thermal properties of the investigated samples. It consisted of three cycles: first heating, cooling, and second heating. The results for all samples are presented in Figure 2. The degree of crystallinity (X_c_) of PLA was calculated using Formula (2), where ΔH_m_ is the melting enthalpy of PLA, x_a_ is the total mass fraction of additives (TCP, PEG and PCL) and ${∆H}_{m}^{o}$ is the theoretical melting enthalpy of 100% crystalline PLA that is equal to 93 J/g [50].

(2) $$X_{C}=\frac{∆H_{m}}{(1-x_{a}{)\cdot H}_{m}^{0}}\cdot 100\%.$$

The DSC results showed that the addition of TCP had an impact on the transformations that occur in polymers. Comparing the curves of pure PLA and samples containing TCP, it can be observed that all of the transitions were shifted and took place at lower temperatures, which may suggest that the presence of TCP affects the structure and may influence the polymer chain mobility. The degree of crystallinity also decreased, indicating that the addition of TCP hinders the crystallization process of PLA. However, the degree of crystallinity increased significantly in the presence of other polymers, although it is still lower than that for pure PLA. For sample 1 (PLA-TCP), the typical transitions of PLA take place during the heating process, such as glass transition, cold crystallization, and melting at 41 °C, 109 °C, and 153 °C, respectively (Table 2). During the second heating cycle, the glass transition with enthalpy relaxation is observed, while cold crystallization does not occur. This may be caused by the presence of TCP particles that interfere with the crystallization of PLA. The addition of PEG (both 600 and 2000) did not significantly change the temperatures of transformations of PLA during the first heating cycle. For sample 2 (PLA-TCP-PEG2000), we could observe the glass transition with relaxation at 51 °C, while during the second heating cycle it was shifted to lower temperatures and occurred at 14 °C. On the other hand, no PEG transformations could be observed during the entire process. This suggests that PEG2000 in sample 2 (PLA-TCP-PEG2000) was present only in the amorphous phase. Taking this into account, we can conclude that PLA was plasticized with PEG2000. However, the endothermic peak that could be observed in sample 2 (PLA-TCP-PEG2000) around 30 °C may indicate the melting of PEG. In the case of sample 3 (PLA-TCP-PEG600), a peak at around 13 °C was visible. This indicates that the melting of PEG600, although the baseline was significantly lowered, suggesting that the glass transition of PLA also occurred. Interestingly, cold crystallization of PLA could be observed in samples containing PCL, while this phenomenon was not visible in sample 3 (PLA-TCP-PEG600), suggesting that PEG600 interfered with the PLA chain. The temperature of glass transition with the relaxation enthalpy of PLA and the melting temperature of PCL are in the same temperature range. That is why on the curves of sample 4 (PLA-TCP-PCL) and 5 (PLA-TCP-PCL-PEG600) we could observe the overlap of these effects with the sharp endothermic peak and the change in baseline position, although cold crystallization and melting of PLA occur without any significant changes.

### 3.3. Morphology and Microstructure of Fabricated Scaffolds

**Figure 3:**
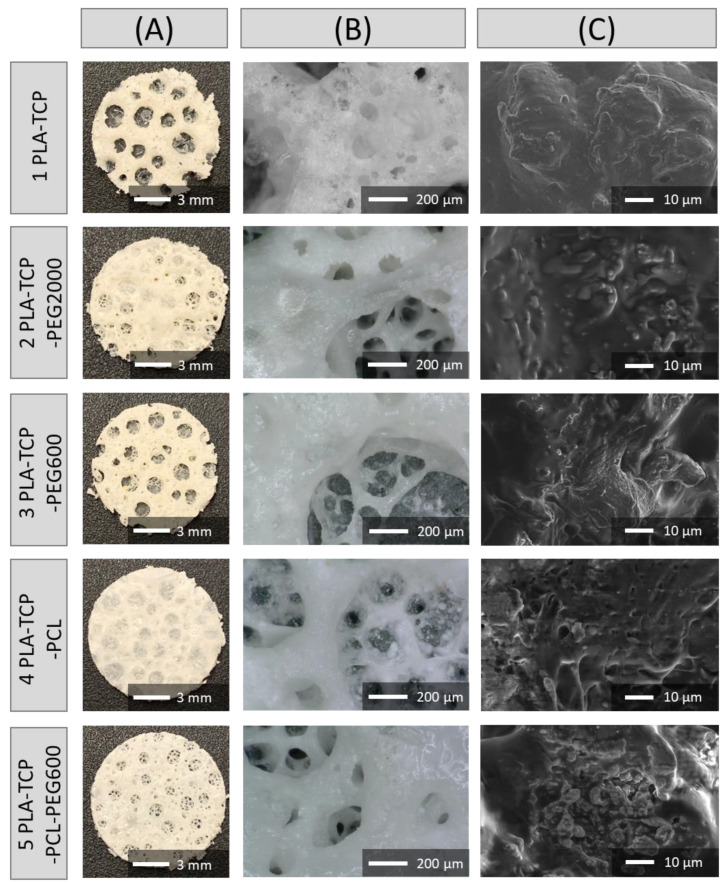
Gross morphology (**A**), optical microphotographs (**B**), and SEM images (**C**) of the obtained samples.

In the camera images (Figure 3A) it can be observed that all samples had a porous structure. This was even better displayed in the optical microscopy pictures (Figure 3B), where, apart from the large open pores, there were many smaller ones to be seen. The surface of the pore walls was rather rough, which could be caused by the addition of TCP particles. To better visualize the microstructure of the samples, scanning electron microscopy (SEM) images were taken (Figure 3C). In each sample, TCP particles embedded in the polymer matrix were observed and their distribution was rather homogeneous. This confirms that the method of preparation allows scaffolds with defined microstructures to be obtained that are characterized by open porosity that varies between 58% and 65%. Compared to sample 1 (PLA-TCP), the addition of different polymers made no statistically significant differences in porosity. Such a hierarchical microstructure mimics the microstructure of spongy bone. The pore size varied between 100 and 1500 µm with a median of less than 300 µm for all samples.

### 3.4. Water Contact Angle Measurement

**Figure 4:**
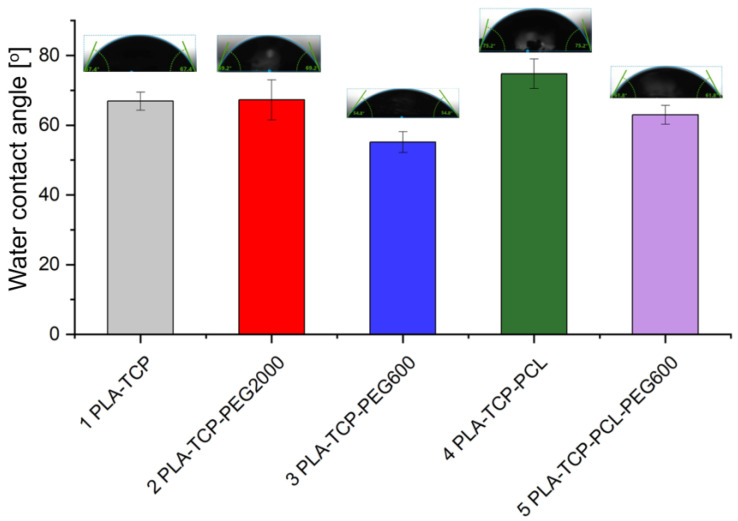
Wettability of samples assessed by the sessile drop method. Above each bar, the representative shape of the droplet is shown.

To characterize the hydrophobic and hydrophilic properties of the scaffolds, we measured the water contact angle. The wettability of the surface of the scaffold is crucial for the adhesion and behavior of the cells. Normally, mammalian cells adhere preferentially to moderately hydrophilic surfaces [51,52]. All water contact angles were within the range of 54.8–75.2° (Figure 4), which indicates that the samples were rather hydrophilic. The lowest contact angle was observed for sample 3 (PLA-TCP-PEG600), while the highest was observed for sample 4 (PLA-TCP-PCL). This suggests that the addition of PEG600 can be used to increase the hydrophilicity of the samples, while the addition of highly hydrophobic PCL can be used to increase the hydrophobicity. The addition of PEG2000 to PLA-TCP-PCL (sample 2) did not appear to have any significant impact on the wettability because the water contact angle was comparable to the value obtained for sample 1, i.e., that containing only PLA and TCP. For sample 5, which consisted of both PEG600 and PCL, the values of water contact angles were also the same as those for control sample number 1.

### 3.5. Degradation Studies

**Figure 5:**
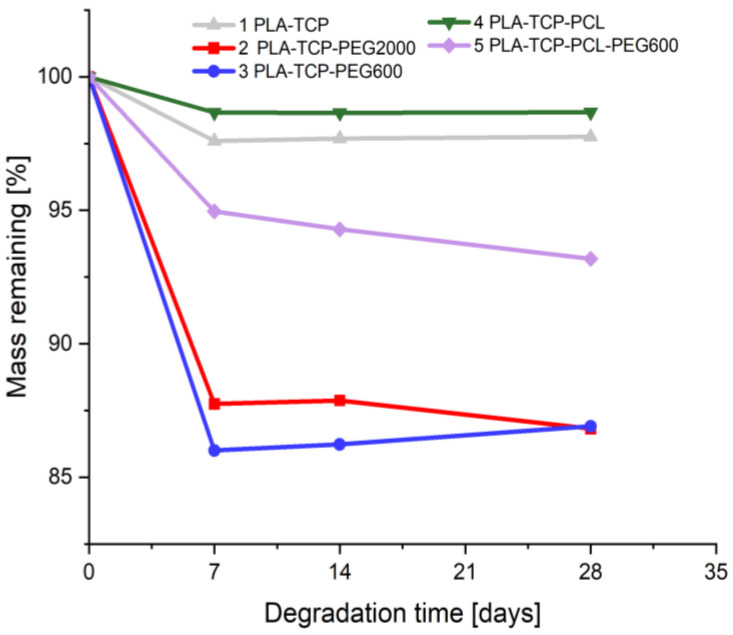
Weight loss of samples during the degradation study in phosphate buffered saline.

**Figure 6:**
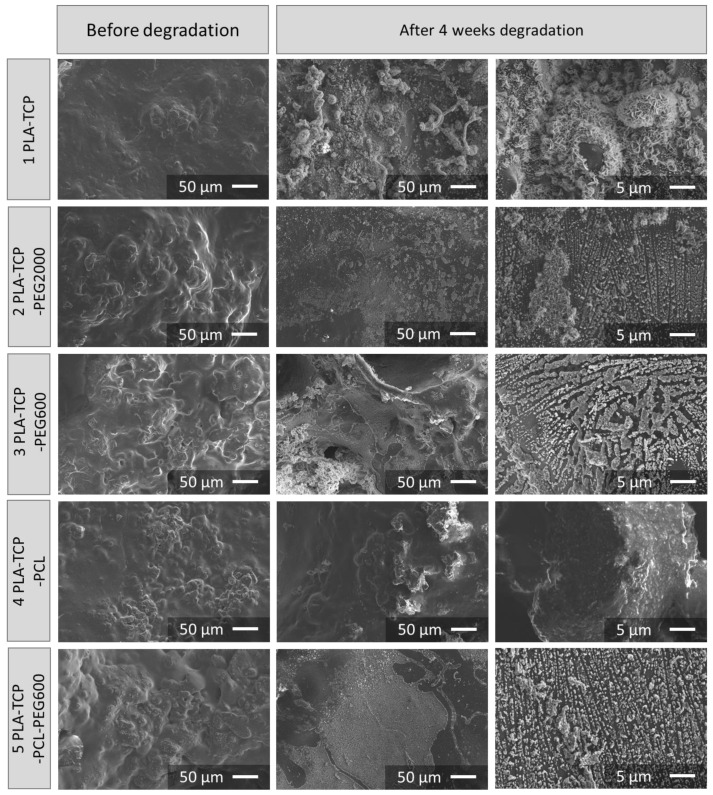
SEM images of samples before and after 4 weeks of degradation in phosphate buffered saline.

To investigate the hydrolytic degradation of the fabricated samples, we exposed them to conditions that mimic physiological fluids of the human body. Briefly, they were immersed in PBS and kept at 37 °C. In a predetermined period of time, the mass of the samples was measured to observe the degradation rate (Figure 5). In addition, SEM pictures were taken before and after the degradation study (Figure 6).

Within the first seven days of the degradation study, the highest mass loss was observed for all samples (Figure 5). For the next 28-day time periods, only slight changes in mass were observed, which were found for each sample, except sample 5 (PLA-TCP-PCL-PEG600). The largest total mass loss was detected for samples containing PEG, i.e., 2 (PLA-TCP-PEG2000) and 3 (PLA-TCP-PEG600), while the lowest was detected for samples 1 (PLA-TCP) and 4 (PLA-TCP-PCL). Based on these results, it can be concluded that the addition of PEG600 or PEG2000 accelerates the degradation process, since the most dynamic mass loss was found for all samples containing this polymer. However, the addition of PCL inhibits the degradation process.

SEM observations (Figure 6, first column) showed that on the surface of the pristine composite samples there were plenty of small bulges, which confirms the presence of TCP particles covered with the polymer or polymer blends. On the SEM images taken of the samples after 4 weeks of incubation in PBS (Figure 6, second column), the polymers were partially degraded and the TCP particles were exposed. Interestingly, TCP particles appeared to be reactive, as inorganic mineral deposits were formed on the surface of all scaffolds, except sample 4 (PLA-TCP-PCL), presumably due to its more hydrophobic nature and lower susceptibility to degradation. At higher magnification (Figure 6, third column) for sample 1 (PLA-TCP), plate deposits were observed. However, on all samples containing PEG (2, PLA-TCP-PEG2000; 3, PLA-TCP-PEG600; and 5, PLA-TCP-PCL-PEG600) round cauliflower deposits were visible. On sample 4 (PLA-TCP-PCL), deposits were not observed.

### 3.6. Biological Performance

#### 3.6.1. Cytotoxicity Tests with Extracts

**Figure 7:**
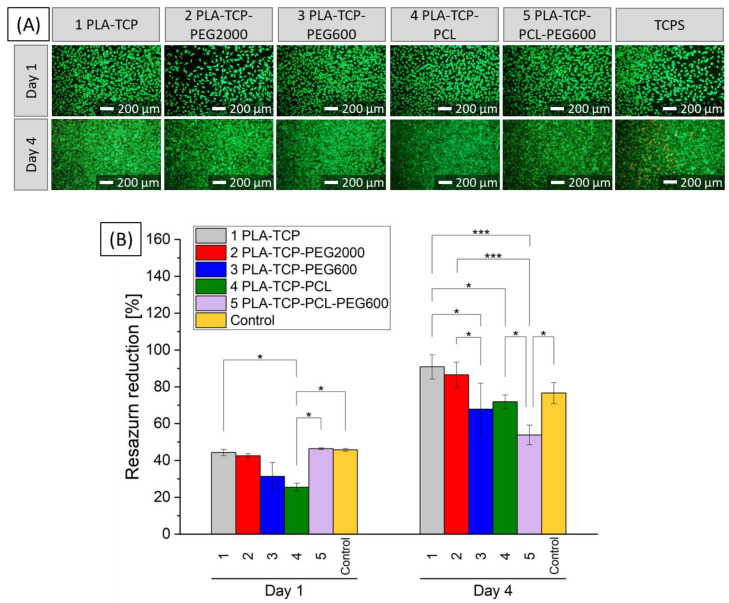
Live/dead staining (**A**) and metabolic activity (**B**) of L929 fibroblast-like cells cultured in the presence of 10% extracts of samples or in DMEM (Control), where *p* * < 0.05, *p* *** < 0.001.

As a first step to assessing whether the samples were cytotoxic, 10% extracts were prepared and placed in contact with cells. After 24 h or 4 days, samples were stained with calcein AM and propidium iodine (live/dead staining) and then observed with a fluorescence microscope. Images of stained cells are shown in Figure 7A. As time passed, the number of cells increased for each sample and only a few dead cells were observed, which is a normal phenomenon. This indicates that the extracts studied cannot be classified as cytotoxic. According to AlamarBlue results (Figure 7B), cells proliferated well in contact with the extracts of all samples. There was no decrease greater than 70% compared to the control sample consisting of DMEM (except for samples 3 and 4 on day 1), which, according to ISO 10993-5, means there was no cytotoxicity [53]. On day 4, when compared to DMEM, a statistically significant difference was visible only for sample 5 (PLA-TCP-PCL-PEG600).

#### 3.6.2. In Vitro Studies with MG-63 Cells

**Figure 8:**
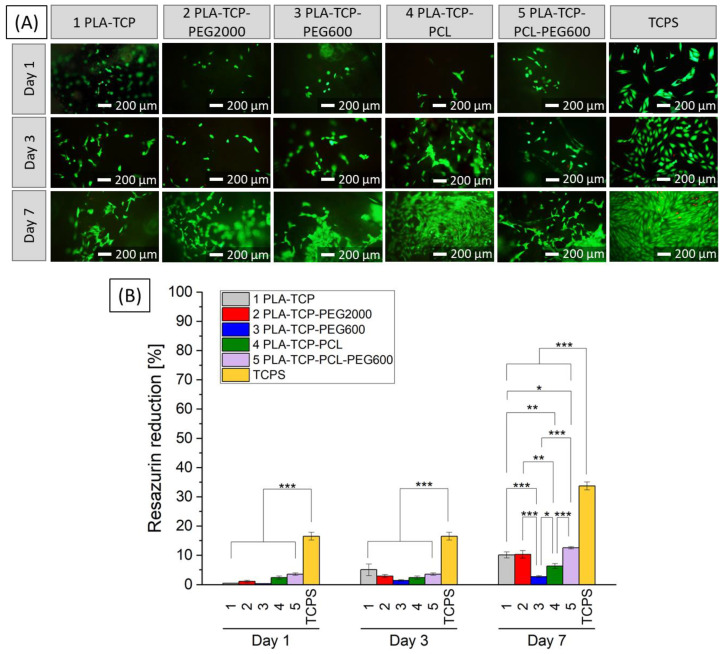
Live/dead staining (**A**) and metabolic activity (**B**) of MG-63 osteoblast-like cells cultured in direct contact with fabricated scaffolds and on TCPS), where *p* * < 0.05, *p* ** < 0.01, *p* *** < 0.001.

MG-63 osteoblast-like cells were cultured in direct contact with the investigated scaffolds to evaluate their cellular responses. On days 1, 3 and 7, cells were stained with calcein AM and propidium iodide (live/dead staining) and observed with a fluorescence microscope (Figure 8A). It was visible that the number of cells was lower when cultured on scaffolds. Furthermore, their morphology differed from the control sample, especially on day 1 when the cells were more rounded, which means that they were poorly attached to the surfaces. As time passed, the cells proliferated on each sample. On day 7, the morphology of the cells in the investigated samples was similar compared to the control flat TCPS, and the number of cells increased. The AlamarBlue results (Figure 8B) showed that the activity of the cells on the scaffolds was much lower and that the difference between all of the evaluated samples and the control TCPS was statistically significant during the entire culture. The greatest increase in activity was visible for samples 1 (PLA-TCP), 2 (PLA-TCP-PEG2000), and 5 (PLA-TCP-PCL-PEG600); however, the activity increased for all scaffolds, meaning that they are not cytotoxic.

## 4. Discussion

PLA is a widely used biodegradable polymer that is approved by the U.S. Food and Drug Administration (FDA). Many researchers have investigated this material, as it exhibits great potential for use in bone tissue engineering [54]. Different forms of this biocompatible polymer are being used, such as microspheres [55,56,57], membranes [58,59,60] or scaffolds [54,61,62,63]. One of the methods used to fabricate porous polymer scaffolds is the gel-casting method [37], which is a simple and effective way to obtain samples with random porosity with a pore size in a range from100 to 1500 µm. The movement of nutrients and cells through porous implants has been reported to be highly influenced by the size of the pores. Larger pores promote blood supply and bone ingrowth, but the strength of the scaffold decreases [9]. Taking this into account, it is important that the scaffold has a hierarchical pore architecture, since the size of the macropores of the cancellous bone varies from 320 to 1670 µm [64].

Different ways of changing the properties of PLA are being used. One of the most common is to blend it with different polymers, such as PCL [65,66] or PEG [16,56,60], which are also approved by the FDA [67,68]. To overcome the bioactivity issue, bioactive substances are also being added to such blends, e.g., bioglass or bioceramic [24,69,70]. The addition of PCL and PEG (both 600 and 2000) did not have any significant impact on the microstructure of the samples, and the TCP particles were visible on each scaffold. The particles were spread quite well in the polymer matrix, although agglomerates also occurred. They appear to be reactive, as after 4 weeks of degradation, calcium phosphate deposits were created on the surfaces of the scaffolds, especially on sample 1 (PLA-TCP). However, the addition of TCP to the matrix changed the thermal properties of the materials. Transitions that occur in the polymer were shifted, indicating that the chain mobility was altered because of the presence of ceramic particles. Moreover, the degree of crystallinity was decreased, which may suggest that TCP particles hinder the crystallization process of PLA. The degradation rate of the scaffolds depended on their composition. The slowest degradation rate was observed for sample 4 (PLA-TCP-PCL), while the greatest weight loss occurred during the degradation study for samples containing PEG (2 and 3). In general, PCL degradation is much slower than PLA, while this is the opposite for PEG that is blended with polymers to increase the degradation rate and improve processability [16,65,68]. That explains the behavior of the investigated samples during the degradation study.

Moreover, the presence of different polymers in the composition of the scaffolds changed the wettability of the materials. The highest water contact angle was observed for sample 4 (PLA-TCP-PCL) and the lowest was for sample 3 (PLA-TCP-PEG600), and these were equal to 54.8° and 75.2°, respectively. This shows that the addition of PCL increased the hydrophobicity, while PEG decreased this parameter. According to the literature, PCL exhibits a more hydrophobic nature compared to PLA, while PEG is very hydrophilic [65,68], which corresponds well to our results. According to the literature, mammalian cells adhere preferentially to moderately hydrophilic surfaces with a water contact angle between 40 and 70° [51]. This suggests that the surface of sample 4 (PLA-TCP-PCL) is slightly too hydrophobic for cells to adhere to. On the contrary, fibroblast adhesion has been reported to be highest when the water contact angle is between 60 and 80° [52]. Taking this into account, sample 3 (PLA-TCP-PEG600) looks too hydrophilic. However, cell adhesion is dependent on other factors, such as topography and roughness [51,52]. Although the presence of different polymers in the blends has affected degradation and wettability, the impact of the blending was not clearly visible for the FTIR study. The FTIR spectra look similar, which means that no new chemical bonds were formed during the fabrication of the scaffolds. Only a double band originating from C=O stretching vibrations around 1750 cm^−1^ confirms the presence of both PLA and PCL in samples 4 (PLA-TCP-PCL) and 5 (PLA-TCP-PCL-PEG600). The thermal properties of the investigated scaffolds changed with the addition of different polymers. The peaks of cold crystallization and the melting of PLA shifted when PEG or PCL were present in the sample. Moreover, the thermal effects of PLA and PCL overlapped, whereas some of the transformations for PEG were not observed. This suggests that PEG occurs in an amorphous phase and acts as a plasticizer. Furthermore, the transition temperature during the first and second heating cycles was different, which means that the scaffold preparation process has an impact on the thermal properties of the samples [71]. As shown, the physicochemical properties of the fabricated scaffolds differed depending on the composition of the blend. A similar situation could be observed in the case of mechanical properties. According to the literature, the addition of PCL to the PLA matrix may enhance the fracture toughness of the base polymer [39,40]. On the other hand, the presence of plasticizer, such as PEG, improves the elasticity of PLA [72,73]. Mechanical properties are crucial features for scaffolds designed for use in bone tissue engineering, and thus further study is required on this topic.

Biological evaluation with L929 fibroblasts cultured in 10% extracts of the samples shows that they are not toxic. Compared to the control sample, a decrease of more than 70% was visible only for sample 3 (PLA-TCP-PEG600) and 4 (PLA-TCP-PCL) on day 1. However, cells proliferated while cultured in each extract and the statistically significant difference on day 4, when compared to TCPS, was observed only for sample 5 (PLA-TCP-PCL-PEG600) and the results showed the lowest increase in metabolic activity for this sample. Interestingly, sample 5 showed great performance during this study with MG-63 osteoblast-like cells cultured in direct contact with the material. According to the literature, surface hydrophilicity may result in better cell attachment [74]. This property can be customized by blending different polymers. It was reported that PEG can improve hydrophilicity of PLA [62]. However, mixing PCL with PLA (with higher hydrophilicity) may enhance the biocompatibility of PCL [75].

The best results with MG-63 cells were observed for samples 1 (PLA-TCP) and 2 (PLA-TCP-PEG2000). Even though PEG has higher hydrophilicity than PLA, the effect of better cell attachment was not observed and there was no statistically significant difference between these samples. However, the metabolic activity of cells for sample 3 (PLA-TCP-PEG600) was significantly lower. That may suggest that the hydrophilicity was already too high and that fewer cells adhered to the surface due to the partial dissolution of PEG600, which is consistent with findings in the literature [52]. The results of sample 4 (PLA-TCP-PCL) were slightly worse compared to samples 1 and 2, which confirms that cell attachment is lower on more hydrophobic surfaces. For sample 5 (PLA-TCP-PCL-PEG600), the cell culture results, as well as wettability, were comparable to samples 1 and 2. However, the metabolic activity of cells increased with culture time for all scaffolds investigated.

Of all five compositions, the best appear to be samples 1 (PLA-TCP), 2 (PLA-TCP-PEG2000), and 5 (PLA-TCP-PCL-PEG2000). They are all thermally stable at temperatures lower than body temperature and are characterized by a similar contact angle, but the degradation rate of each differs significantly. L929 fibroblasts proliferated slightly worse in the extract of sample 5, but the three best samples demonstrated great performance in direct contact with MG-63 osteoblast-like cells with no statistically significant differences on days 1 and 3. Furthermore, the only significant difference between these materials was observed on day 7 between samples 1 (PLA-TCP) and 5 (PLA-TCP-PCL-PEG2000). Thus, depending on the requirements, it is possible to control the degradation rate of the material by changing the composition without significant effects on cytocompatibility.

The porosity of the proposed blends is in the range of 58 to 65%; however, a higher porosity is more suitable for cell interaction [76]. Further study is needed to control the pore formation process more effectively to overcome this limitation. Even though the properties of the fabricated samples need to be more thoroughly investigated, the samples exhibit good cytocompatibility and appear to meet the criteria for application in bone tissue regeneration.

## 5. Conclusions

In this study, we aimed to fabricate polymer scaffolds using a gel-casting technique combined with rapid heating. To do so, we blended PLA with PEG600, PEG2000, and PCL to evaluate the influence of these polymers on the performance of the scaffold. Moreover, to improve the bioactivity of the scaffolds, TCP particles have been embedded in each blend. They can be regarded as bioactive because calcium phosphate deposits are created on the surface during contact with PBS. The samples were characterized by porosity between 58% and 65% and pore size between 100 µm and 1500 µm. The addition of different polymers significantly affected the degradation rate, wettability, and thermal properties of the scaffolds. The susceptibility to degradation decreased for scaffolds containing PCL because of its higher hydrophobicity. The presence of PEG accelerated scaffold degradation because of its hydrophilicity and water solubility. The extracts of all scaffolds were not cytotoxic to L929 fibroblasts. The scaffolds supported the adhesion and proliferation of osteoblast-like MG-63 cells while cultured in direct contact. Samples 1 (PLA-TCP), 2 (PLA-TCP-PEG2000), and 5 (PLA-TCP-PCL-PEG2000) appear to best meet the demands for application in bone tissue; however, further studies regarding mechanical properties and microbial tests are required. Depending on the size of the defect and its location in the body, it is possible to create a scaffold with an appropriate degradation rate without affecting the adhesion of the cells to the surface.

To sum up, the presented method of fabrication is effective in obtaining highly porous, bioactive composite scaffolds with a defined microstructure and degradation rate that can be controlled and adapted to the medical needs.

## Figures and Tables

**Table 1:** Percentage composition of the produced scaffolds.

Sample	Component [wt.%]				
	PLA	TCP	PEG600	PEG2000	PCL
1 PLA-TCP	72	28	-	-	-
2 PLA-TCP-PEG2000	60	28	-	12	-
3 PLA-TCP-PEG600	60	28	12	-	-
4 PLA-TCP-PCL	60	28	-	-	12
5 PLA-TCP-PCL-PEG600	50	28	10	-	12

**Table 2:** Glass temperature (T_g_), cold crystallization temperature (T_cc_), and melting temperature (T_m_) of PLA-based materials and degree of crystallinity (X_c_) of PLA.

Sample	T_g_ [°C]		T_cc_ [°C]		T_m_ [°C]		X_c_ [%]	
	1st Heating	2nd Heating	1st Heating	2nd Heating	1st Heating	2nd Heating	1st Heating	2nd Heating
PLA	66	61	99	102	170	168	58	55
1 PLA-TCP	41	54	109	^nd^	154	146	27	3
2 PLA-TCP -PEG2000	51	14	80	89	155	130	39	35
3 PLA-TCP -PEG600	46	42	^nd^	^nd^	152	143	39	17
4 PLA-TCP -PCL	^nd^	^nd^	97	124	56 (PCL) 153 (PLA)	57 (PCL) 153 (PLA)	26	22
5 PLA-TCP-PCL-PEG600	^nd^	35	^nd^	77	154 (PLA)	56 (PCL) 152 (PLA)	37	35

^nd^—not determined.

## Data Availability

Data will be provided upon request.
